# The effects of mepiquat chloride (DPC) on the soluble protein content and the activities of protective enzymes in cotton in response to aphid feeding and on the activities of detoxifying enzymes in aphids

**DOI:** 10.1186/s12870-022-03597-7

**Published:** 2022-04-26

**Authors:** Quan-Cheng Zhang, Xiao-Xia Deng, Jun-Gang Wang

**Affiliations:** College of Agriculture, Shihezi University, Shihezi, 832003 China

**Keywords:** Mepiquat chloride (DPC), Cotton, Aphids, Protective enzymes, Detoxifying enzymes, Resistance ability

## Abstract

**Background:**

Mepiquat chloride (DPC) enhances the resistance of cotton plants, and it is widely used as a growth regulator. DPC can stimulate photosynthesis, stabilize the structure of cotton leaves, and affect population reproduction and energy substances in *Aphis gossypii* Glover (cotton aphids), but interactions between DPC and cotton aphids remain unclear. In this study, we analyzed the physiological responses of cotton to DPC, and the toxicity of DPC toward cotton aphids, before and after feeding, to explore the DPC-induced defense mechanism against cotton aphids.

**Results:**

Measurements of protective enzyme activity in cotton showed that the soluble protein contents, peroxidase (POD) activity, and catalase (CAT) activity in cotton treated with different concentrations of DPC were higher than in the control. Superoxide dismutase (SOD) activity was higher than that of the control when the concentration of DPC was < 0.1 g/L. Under aphid feeding stress, POD activity in cotton treated with a low insect population density was significantly lower than in the controls, but the reverse was true for cotton treated with a high insect population density, and SOD activity was positively correlated with population density. The activities of detoxification enzymes in field and laboratory experiments showed that DPC promoted the specific activity of glutathione S-transferase (GST) in cotton aphids, while the specific activities of carboxylesterase (CarE) and acetylcholinesterase (AchE) were decreased.

**Conclusions:**

DPC enhanced the aphid resistance in cotton by increasing the soluble protein content and the activity of protective enzymes. It also had a toxic effect on cotton aphids by increasing GST activity (the main DPC target). DPC increased the soluble protein content and protective enzymes activity in cotton under aphid stress, and thereby enhanced tolerance to cotton aphids. It conclude that DPC interferes with cotton aphids through indirect (DPC induced cotton defense responses) and direct (DPC toxicity to cotton aphids) ways, which plays a positive role in interfering with cotton aphids.

## Background

Cotton is an important economic crop in many places in the world, and cotton plants have an indeterminate growth habit. As long as the environmental conditions are suitable, it can continue squaring, flowering, and producing bolls [1], which leads to a balance between reproductive growth and vegetative growth [2]. An excessive nutrient supply leads to poor reproductive organ development, which in turn results in low yield and poor fiber quality [3]. Therefore, in the cultivation and planting of cotton, the grower should not only focus on the management of water and fertilizer in the field [4, 5], but should also consider the use of exogenous plant hormones for chemical regulation [6] for reasonable control of cotton growth and development. This will determine the shape of the ideal plant type [7] by reducing plant height [8], shorten the main stem and fruiting branch internodes [9, 10], improve light transmittance, reduce boll shedding [11], and promote boll opening [12] to improve economic yield [13, 14].

Mepiquat chloride (1,1-dimethylpiperidium chloride; DPC) is a synthetic plant hormone that is widely used as a growth retardant in cotton [15–18]. DPC inhibits gibberellin synthesis in plants and can control cotton vegetative growth [19, 20], reduce boll shedding, and promote boll development and root growth in cotton [10, 21, 22]. DPC has also been shown to improve cell membrane stability and stress tolerance in plants [23, 24]. Our previous research results also helped to explain the action of DPC. Treatment with appropriate concentrations of DPC can increase the content of free proline and soluble protein in cotton leaves, reduce the malondialdehyde (MDA) content, increase the osmotic pressure resistance of cotton cells, promote stress resistance, and improve drought resistance in cotton [25, 26]. By increasing leaf wax deposition and chlorophyll content, the photosynthetic rate of cotton leaves was effectively improved [27]. In addition, DPC induces the production of phenolic secondary metabolites in cotton leaves, and the increase in lignin, total phenols, tannins, flavonoids, and related compounds can enhance stress resistance in cotton [28].

Science-based, rational application of DPC can not only promote high yields in cotton, but can also enhance the defense response of host plants to pests [29]. Studies have shown that DPC has a negative effect on the growth and development of the cotton pests *Helicoverpa armigera* (Hübner) (cotton bollworm) and *Tetranychus cinnabarinus* (Boisduval) (carmine spider mite), and with increasing DPC concentrations, the body weights of *H*. *armigera* larvae reared in the laboratory decreased in an 'S' curve (sigmoid function), which may be due to the large increase in the amount of gossypol produced by the cotton plants [30]. At the same time, the use of DPC can block feeding in *T*. *cinnabarinus*, leading to reproductive decline [31]. DPC has a 'Dual Regulation Effect' on cotton growth and development, which makes it a key chemical regulator in the cotton planting industry [32].

The cotton aphid, *Aphis gossypii* (Glover), is the main pest of cotton. *A*. *gossypii* can cause damage throughout the cotton growth period, which can cause wilting and deformity in cotton seedlings [33]. The honeydew secreted by cotton aphids also affects photosynthesis and fiber quality in cotton, and can seriously endanger cotton production [34]. In the process of artificial control of *A.* gossypii, the cotton aphids are forced to participate in this process. We have previously found that cotton aphids need to consume energy reserves in the form of body fat to cope with the stress associated with exposure to DPC. At the same time, carbohydrates, free amino acids, and protein energy sources begin to accumulate in cotton aphids to maintain their normal life activities [35]. In response to DPC stimulation, the specific activities of SOD, POD, and CAT increase rapidly [36]. With increases in DPC treatment time, adult longevity and reproductive capacity in cotton aphids decreased gradually, and DPC had a good inhibitory effect on the cotton aphid population [37].

Although the results of our previous studies have shown that DPC can induce the synthesis of primary and secondary metabolites and enhance photosynthesis in cotton to improve stress resistance [25–28], DPC also interferes with the physiological metabolism and growth and development of cotton aphids [35–37]. However, these studies only focused on the relationships between DPC and cotton aphids, DPC and cotton. In field applications, we often faced the DPC-cotton-cotton aphid interaction. However, this relationship has yet to be studied. Based on previous studies, we hypothesize that DPC induces defense responses against cotton aphids, and DPC also directly affects cotton aphids, which plays a positive role in interfering with cotton aphids. Therefore, this study focused on (1) the effects of DPC on protective enzyme activity in the cotton plant, (2) the mechanism of DPC toxicity on cotton aphids, (3) as mediated by DPC treatment, the physiological response of cotton to cotton aphids before and after feeding on cotton. The results of our study will uncover the nature of the DPC-cotton-cotton aphid interaction and clarify the role that DPC plays in the defense response of cotton to aphid feeding, and will provide a theoretical basis for the science-based application of DPC to cotton in the field.

## Methods

### Experimental site and plant materials

The experimental field was in the Test Site of Shihezi University (86º E, 44º N). The cotton variety used in this field experiment was ‘New Upland Early Maturity 44’. DPC was purchased from Anyang Xiaokang Pesticide Co., Ltd (Anyang city, China).

The cotton aphids came from an artificial breeding population maintained in the insect greenhouse of Shihezi University. Over 30 generations of aphids have been subcultured on cotton. Feeding temperature was 26 ± 1 °C, relative humidity was 60–80%, and the light intensity was 9,000 lx with a 14 h light:10 h dark photoperiod.

### Determination of protective enzyme activity in cotton induced by DPC treatment

At the flowering and boll-forming periods, cotton plants were sprayed with DPC at concentrations of 0, 0.05, 0.1, 0.25, 0.5, and 1.0 g/L (30 kg water/667m^2^) using a completely randomized block design. The changes in soluble protein contents and POD, SOD, and CAT activities in cotton were determined at 5, 10, 15, and 20 days after application. There were three replicates per treatment.

### Determination of detoxifying enzyme activity in aphids following DPC treatments in the field

During the flowering and boll-forming periods, cotton plants were selected randomly for the experiments. The plants were infested with 300 test aphids on the top three leaves and covered with gauze (0.5 m * 1.2 m, 120 mesh). One day after infestation, the plants were sprayed with DPC at five concentrations (0, 0.05, 0.1, 0.25, 0.5, and 1 g/L). At 5, 10, and 15 days after application, 100 wingless adults were selected to determine the specific activities of AchE, CarE, and GST. Each treatment was repeated three times.

### Determination of detoxifying enzyme activity by DPC leaf immersion treatment

Cotton leaves soaked in different concentrations of DPC were placed on their abaxial surfaces in 10 cm petri dishes containing 5 mL agar liquid, and 30 wingless aphids were collected with a brush and placed on the cotton leaves. Defatted cotton wool was used to surround the cotton leaves to prevent aphids from escaping. After five days of feeding, 100 aphids were selected to determine the specific activities of AchE, CarE, and GST. There were 18 dishes per treatment, and each treatment was repeated three times.

### Determination of cotton protective enzyme activity by DPC treatment under aphid stress

Based on the degree of damage due to cotton aphid feeding, the population density of cotton was divided into three categories; 0 aphids, low population density (100–300 aphids), and high population density (> 500 aphids). After selecting the appropriate cotton plant marker, cotton plants were covered with gauze (0.5 m*1.2 m, 120 mesh), and sprayed with different concentrations of DPC. The changes in soluble protein contents and POD, SOD, and CAT activities in cotton were measured at 5, 10, 15, and 20 days after DPC application. Each treatment was repeated three times.

### Determination of protective/detoxifying enzyme activity

Soluble protein contents were determined by the G-250 dye colorimetric method [38]. POD activity was determined by the guaiacol method. The activity of SOD was determined by the inhibition photoreduction method, where the amount of the enzyme required to inhibit NBT reduction by 50% is defined as a unit of enzyme activity. CAT activity was determined by the UV absorption method. For detailed descriptions of these methods of determination, refer to the publication of Li [39]. The specific activities of AchE, CarE, and GST were determined as described by Wang [40].

### Statistical analysis

Microsoft Excel 2010 was used for data processing and plotting. SPSS 19.0 data processing software was used for statistics, and the mean values and standard errors were calculated. Duncan’s new multiple range test method was used to test the significance between the differences in the means.

## Results

### Effect of DPC dosage on protective enzyme activities in cotton during the flowering and boll-forming period

**Fig. 1:**
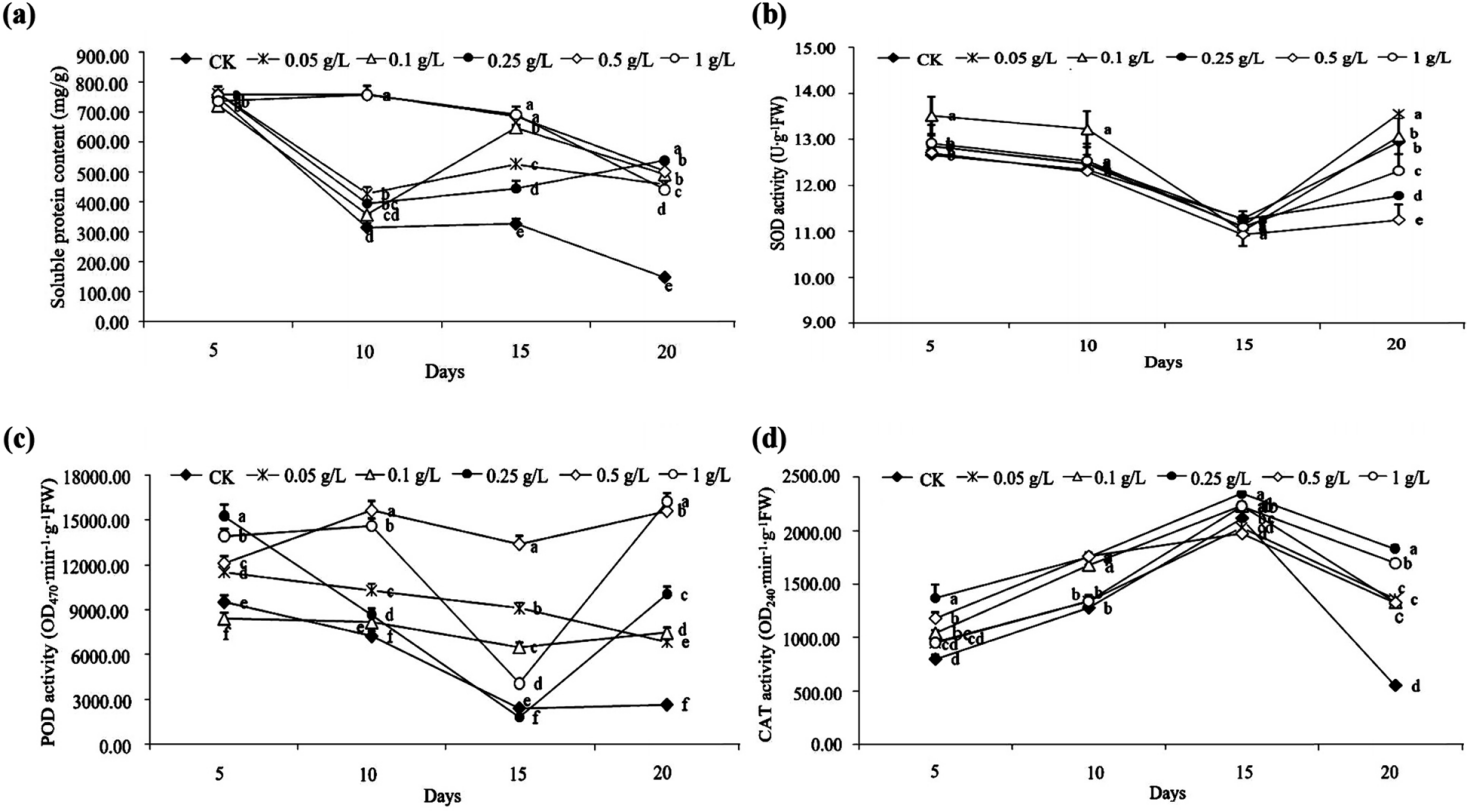
The effects of mepiquat chloride (DPC) treatment on the soluble protein contents and the activities of three antioxidant enzymes in cotton. **a** Soluble protein contents. **b** SOD (superoxide dismutase) activity. **c** POD (peroxidase) activity. **d** CAT (catalase) activity. Lower-case letters indicate significant differences between the control (CK) and the five DPC treatments at different concentrations from 5 to 20 days after DPC application

In this experiment, the soluble protein content and the POD and CAT activities in cotton for each DPC concentration treatment were higher than those of the control. At 20 days after treatment, the soluble protein content, POD activity, and CAT activity in cotton plants sprayed with 0.25 g/L DPC were significantly higher than in the control by 2.60-, 2.33-, and 2.78-fold (*P* < 0.05; Fig. 1a, c, and d). When the DPC concentration was less than or equal to 0.5 g/L, the SOD activity decreased as the DPC concentration increased, after which it increased. The SOD activity in the 0.5 g/L DPC treatment was the lowest, and it was significantly lower than the control by 13.13% (*P* < 0.05; Fig. 1b).

However, at 20 days after DPC treatment, the SOD and CAT activities in cotton showed the opposite trend compared with the early stage of the experiment (SOD activity increased and CAT activity decreased), which may be due to the gradual decrease in DPC efficacy over time (Fig. 1b and d). Therefore, we conclude that spraying with DPC at a concentration of 0.25 g/L during the flowering and boll-forming period can most enhance stress resistance in cotton, and DPC can be applied again 20 days later to regulate cotton growth.

### Effect of DPC treatment on detoxifying enzyme activities in cotton aphids

**Fig. 2:**
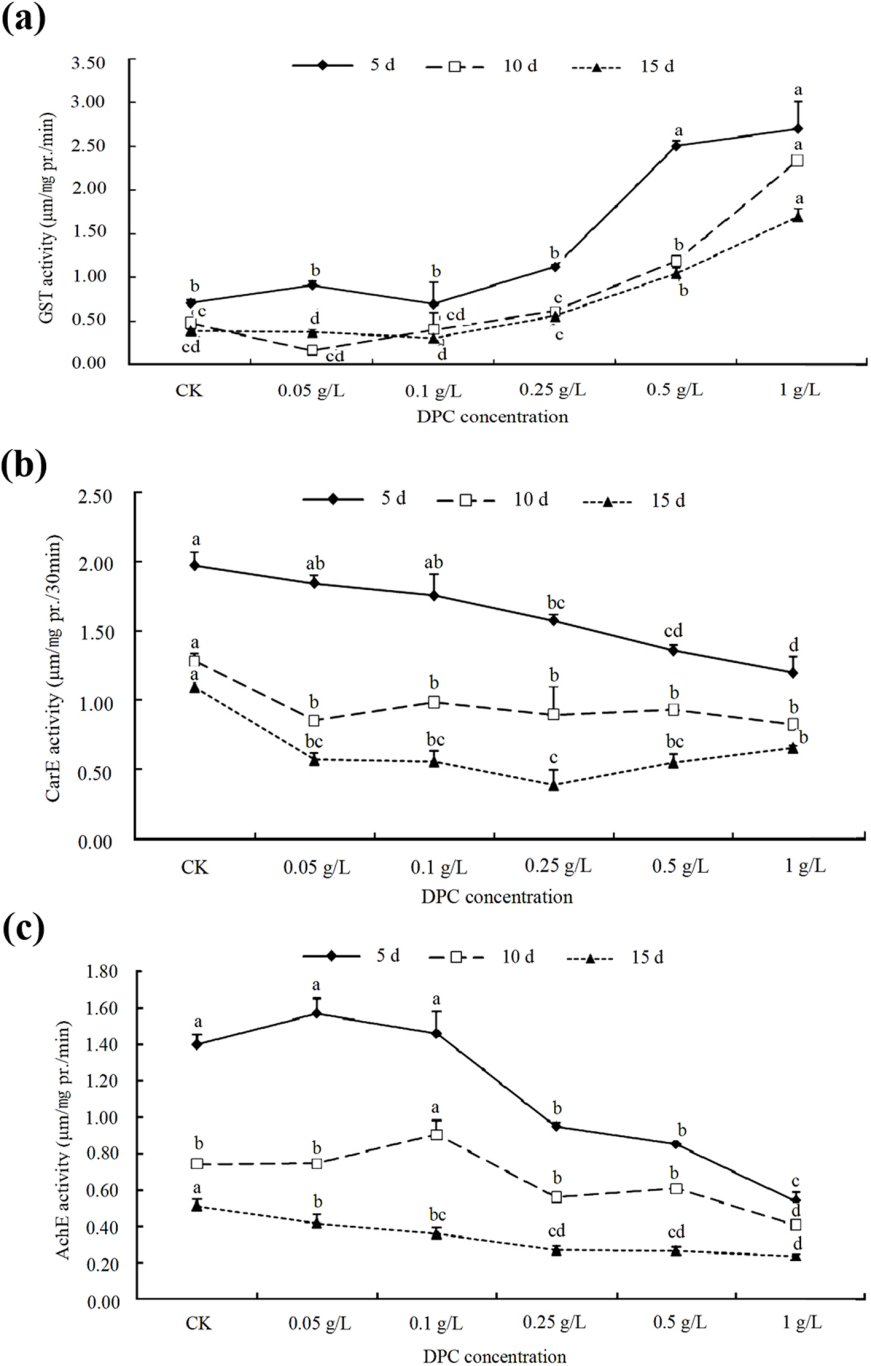
Effects of DPC treatments on detoxification enzyme activity in cotton aphids. **a** GST (glutathione S-transferase) activity. **b** CarE (Carboxylesterase) activity. **c** AchE (Acetylcholinesterase) activity. Lower-case letters indicate significant differences between the control (CK) and the five DPC concentrations after 5, 10, and 15 days of feeding

The specific activity of GST in cotton aphids was increased by spraying the plants with DPC in the field, and GST activity was positively correlated with DPC concentration. With increasing treatment duration, GST activity in cotton aphids decreased, which may be due to enhanced adaptation of cotton aphids to DPC (Fig. 2a). However, the activities of CarE and AchE decreased with increasing DPC concentration (Fig. 2b and c).

**Fig. 3:**
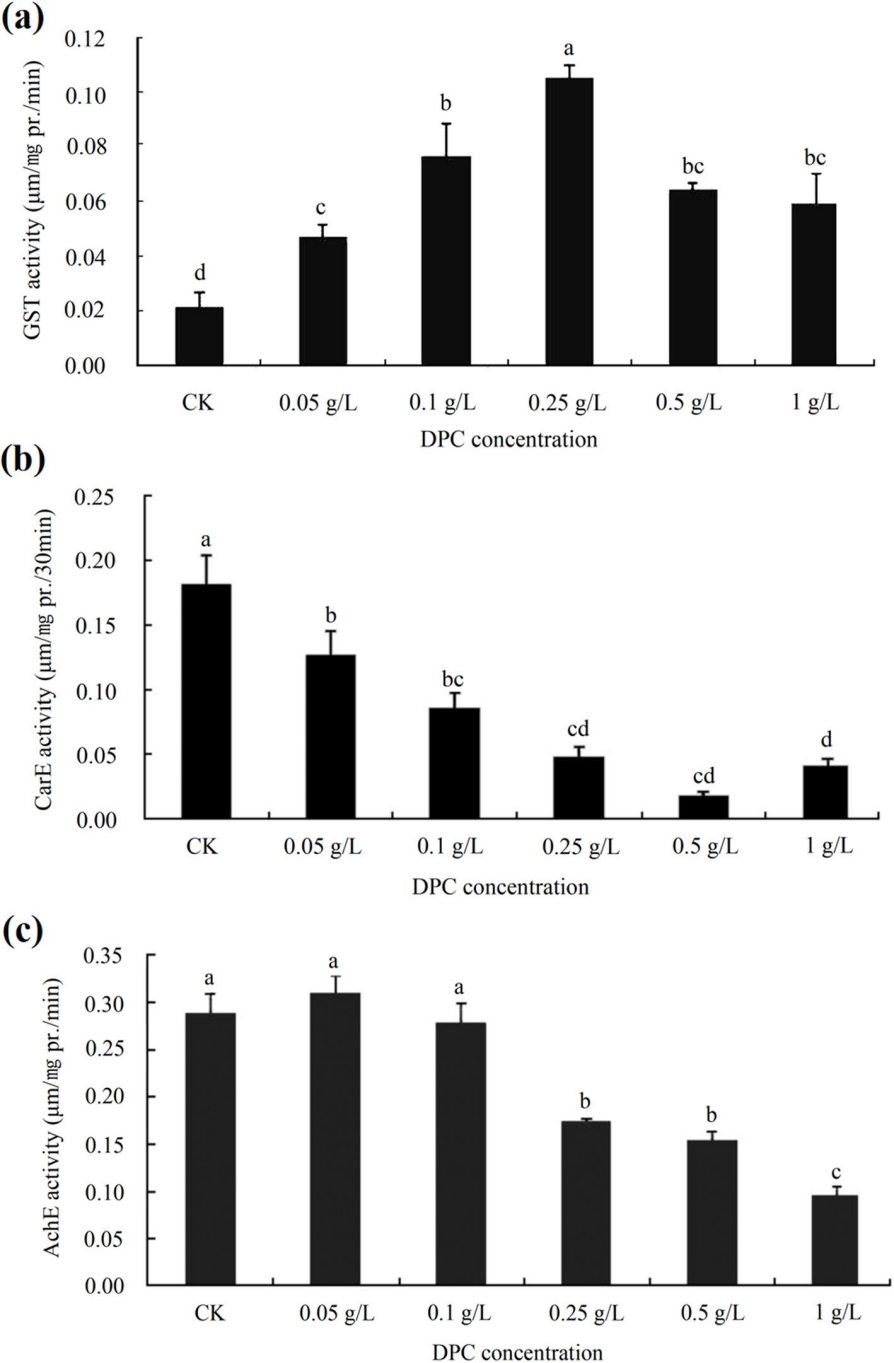
Effects of DPC treatment on the activities of three detoxifying enzymes in cotton aphids. **a** GTS (glutathione S-transferase). **b** CarE (Carboxylesterase). **c** AchE (Acetylcholinesterase). Lower-case letters indicate significant differences between the control aphids (CK) and the aphids on cotton leaves treated with five concentrations of DPC after 5 days of feeding

The leaf dipping test showed that when the DPC concentration was ≤ 0.25 g/L, the GST activity in cotton aphids was significantly higher than in the control and the 0.05 g/L and 0.1 g/L DPC treatments. When the DPC concentration was > 0.25 g/L, the specific activity of the enzyme decreased as the DPC concentration increased, although it remained significantly higher than in the control. Therefore, it appears that DPC concentration ≤ 0.25 g/L has a certain toxicity to cotton aphids, and that the sensitivity of cotton aphids to DPC decreased at the two higher concentrations used in the experiment (Fig. 3a). When the DPC concentration was ≤ 0.5 g/L, the specific activity of CarE decreased with increasing DPC concentration (Fig. 3b). The specific activity of AchE decreased significantly as the DPC concentration increased from 0.05 g/L to 1 g/L (Fig. 3c).

DPC mainly affects the GST activity in cotton aphids, but not the other detoxification enzymes assayed in this study. Therefore, even though DPC acts as a plant growth regulator, when the DPC concentration < 0.5 g/L, the growth of cotton aphids will also have a certain impact, and treatment with higher DPC concentrations will reduce the sensitivity of cotton aphids.

### Effects of DPC on protective enzyme activities in cotton under aphid stress during the flowering and boll-forming period

**Fig. 4:**
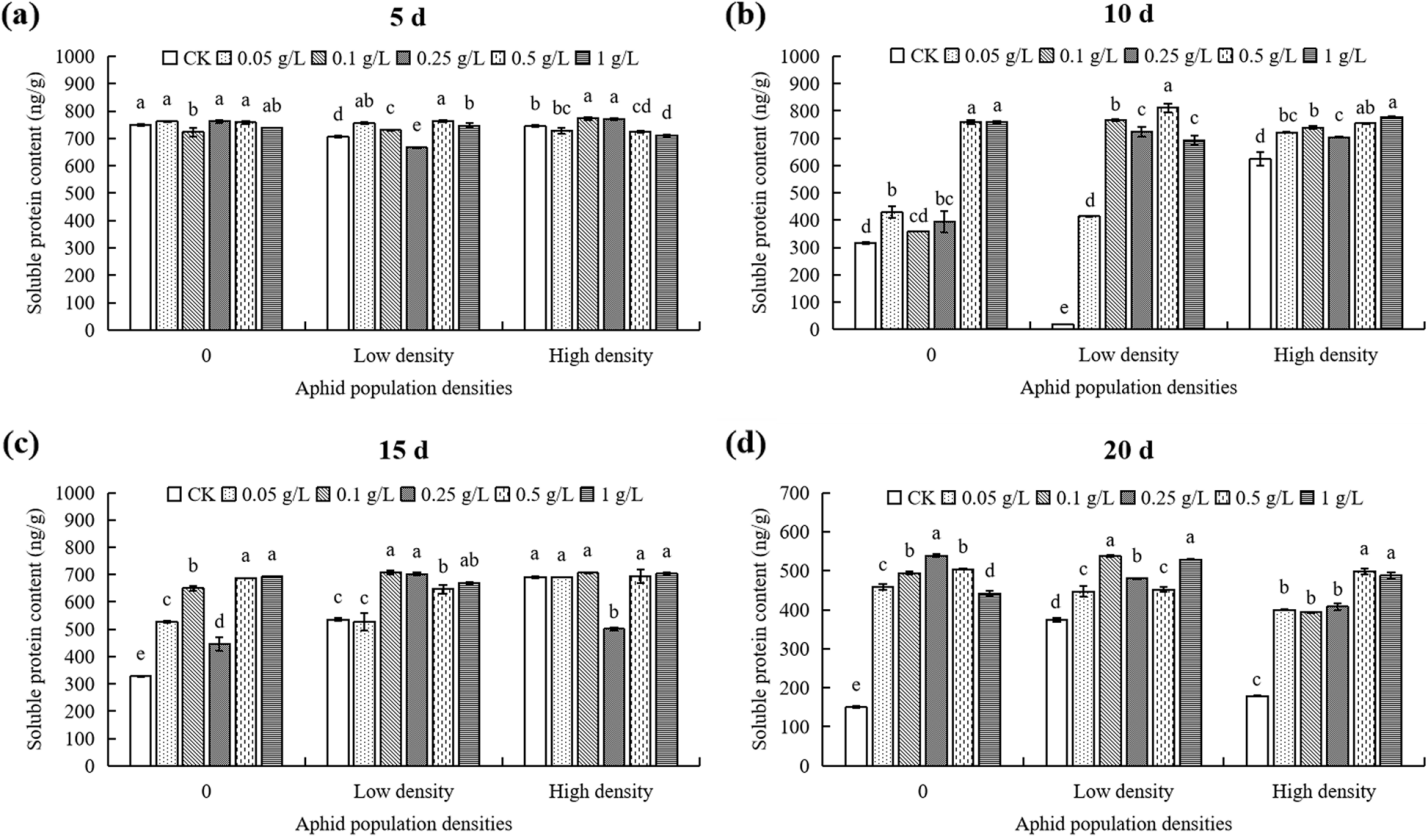
Effects of DPC treatment on the soluble protein content in cotton in response to aphid feeding stress at 5 (**a**), 10 (**b**), 15 (**c**), and 20 d (**d**) after spraying with DPC. Lower-case letters indicate significant differences between the control (CK) cotton plants and the plants in the five DPC treatments at the three aphid population densities

**Fig. 5:**
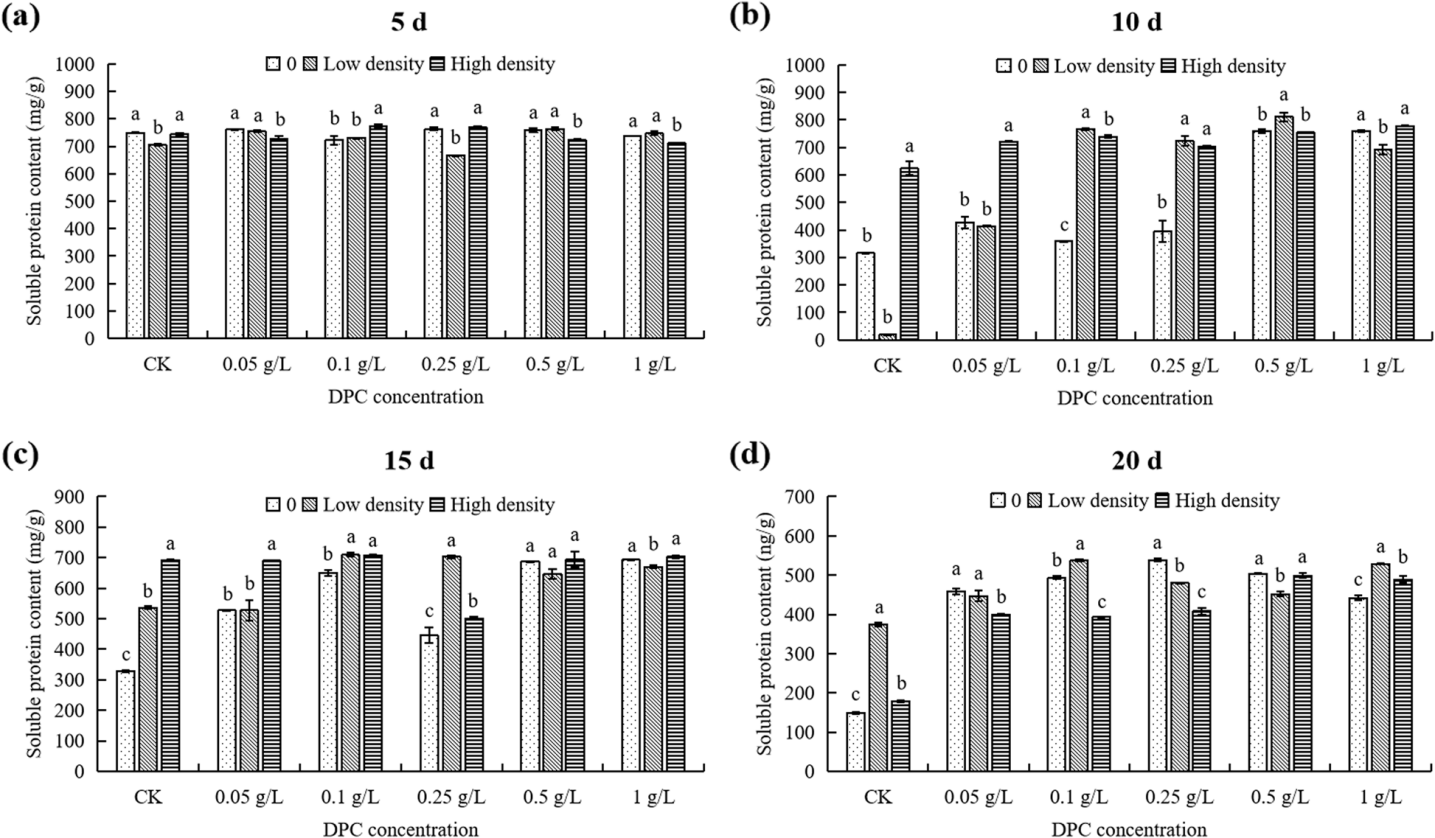
Effects of DPC treatment on the soluble protein content in cotton at 5 (**a**), 10 (**b**), 15 (**c**), and 20 d (**d**) after spraying with DPC. Lower-case letters indicate significant differences between the cotton plants infested with aphids at three population densities for the five DPC concentrations

At 5th day, under low density, the cotton soluble protein content of the 0.25 g/L DPC treatment was the lowest, significantly lower (5.57%) than that of controls (*P* < 0.05; Fig. 4a). Under high population density stress, the soluble protein content of cotton treated with 0.1 g/L DPC was the highest, significantly higher (3.93%) than that of controls (*P* < 0.05; Fig. 4a). At the same DPC concentration, without aphids and under low density, the soluble protein in the content of 0.1 g/L and 0.25 g/L DPC treatments reached minimum values respectively, which were 722.68 mg/g and 666.28 mg/g, respectively (Fig. 5a). Under high density, the soluble protein content in cotton reached a maximum of 773.08 mg/g when treated with 0.1 g/L DPC (Fig. 5a).

At 10th day, under different aphid densities, differences in cotton soluble protein content between different DPC treatments were increased (Fig. 4b). Under low density, the soluble protein content of the 0 g/L DPC treatment was only 18.28 mg/g, significantly lower than that of cotton treated with DPC (Fig. 4b). The soluble protein content of the 0.5 g/L DPC treatment reached the maximum value (811.08 mg/g) in the low density population, which was significantly higher than that of cotton without aphid (*P* < 0.05; Fig. 4b). When the DPC concentrations at 0.1 and 0.25 g/L, the soluble protein content in cotton was significantly affected by low and high density (*P* < 0.05; Fig. 5b).

At 15th day, without aphids, the soluble protein content of cotton in the 0.5 g/L and 1 g/L DPC treatments showed no significant differences, while the other treatments was significantly higher than in the controls (Fig. 4c). Under low density, the soluble protein content in the ≥ 0.1 g/L DPC treatments was significantly higher than in the controls, while in the 0.05 g/L DPC treatment showed no significant differences (Fig. 4c). At the same concentration of DPC, the effect of feeding stress on cotton soluble protein content was not significant at 0.5 g/L DPC, while the effect of other DPC concentrations on soluble protein content was significant (*P* < 0.05; Fig. 5c).

At 20th day, under different aphid densities, the soluble protein content in cotton with DPC treatment were significant higher than that of controls (Fig. 4d). Under low density the soluble protein content at 0.1 g/L DPC was significantly higher than that without DPC treatment (537.48 mg/g) (Fig. 4d). Under high density, the soluble protein content of cotton increased gradually with increasing DPC concentration (Fig. 4d). Also, there were no significant differences in soluble protein content between the 0.05, 0.1, and 0.25 g/L treatments, but it was significantly higher than that of cotton without DPC treatment (Fig. 4d). At a given DPC concentration, the soluble protein content of cotton was significantly affected by the different aphid densities (Fig. 5d). When the DPC the concentration at 0 g/L, the soluble protein content without aphids was the lowest (149.75 mg/g) (Fig. 5d). When the DPC the concentration at 1 g/L, the soluble protein content in cotton with low and high density were higher than without aphids (*P* < 0.05; Fig. 5d).

**Fig. 6:**
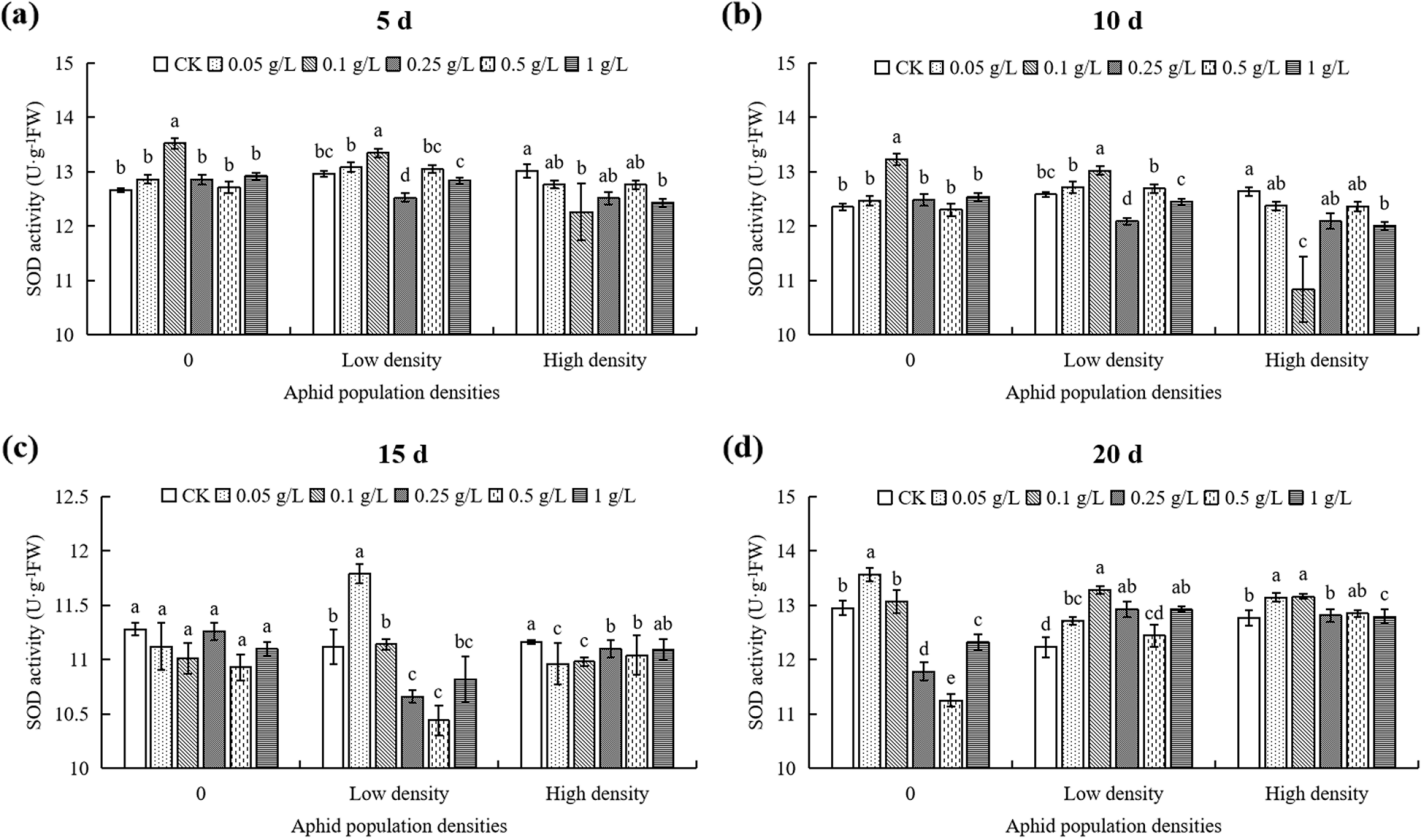
Effects of DPC treatment on SOD (superoxide dismutase) activity in cotton plants infested with aphids at three populations densities at 5 (**a**), 10 (**b**), 15 (**c**), and 20 d (**d**) after spraying with DPC. Lower-case letters indicate significant differences between the control (CK) and the cotton plants in the five DPC treatments at the three aphid population densities

**Fig. 7:**
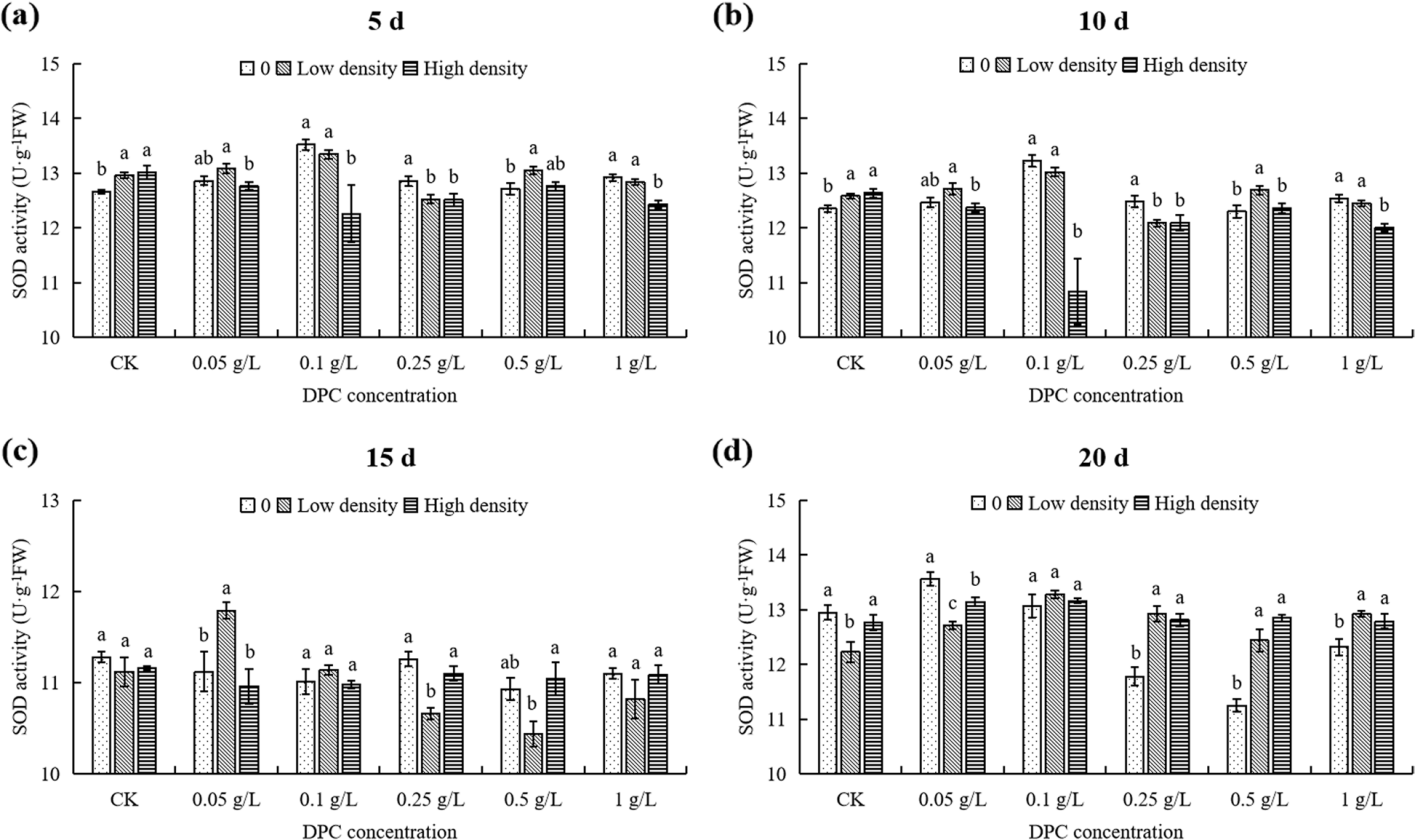
Effects of DPC treatment on SOD (superoxide dismutase) activity in cotton at 5 (**a**), 10 (**b**), 15 (**c**), and 20 d. (**d**) after spraying with DPC. Lower-case letters indicate significant differences between the cotton plants infested with aphids at three population densities for the five DPC concentrations

At 5th day, the SOD activity in cotton without aphids treated with 0.1 g/L DPC treatment was the highest, and was significantly higher (6.79%) than that of controls (*P* < 0.05; Fig. 6a). Under high density, the SOD activity of cotton treated with 0.1 g/L DPC was the lowest, significantly lower (5.76%) than that of controls (*P* < 0.05; Fig. 6a). When the DPC concentration at 0, 0.1, 0.25, and 1 g/L, there were significant difference in SOD activity between the without aphids and the high density treatment, but have no significant difference in the 0.05 g/L and 0.5 g/L DPC (Fig. 7a).

At 10th day, the SOD activity without aphids and 0.1 g/L DPC treatment was significantly different from that of the teratments, and the activity of SOD was the highest (13.23 U·g^−1^FW) (Fig. 6b). When the DPC concentration at 0.25 g/L, the SOD activity in cotton with low and high density were significantly lower than without aphids (*P* < 0.05; Fig. 7b). In the 0.1 g/L DPC treatment, the SOD activities in the control and plants infested with low aphid population densities were not statistically different, but they were significantly different from the SOD activities in plants infested at high population densities (*P* < 0.05; Fig. 7b).

At 15th day, at low aphid population density, the SOD activity of cotton treated with 0.05 g/L DPC was the highest (11.79 U·g^−1^ FW), but there was no significant difference compared with controls (*P* > 0.05; Fig. 6c), and the SOD activity in the 0.5 g/L DPC treatment was only 10.44 U·g^−1^ FW, which was not significantly different from that of the controls (*P* > 0.05; Fig. 6c). There was no significant difference in SOD activity between the no-aphid cotton plants and those infested with aphids at high density for the different DPC concentrations (Fig. 7c). At a DPC concentration of 0.05 g/L, the SOD activity in cotton infested with aphids at a low population density was significantly higher than that in both the no-aphid control and the high population density treatment (Fig. 7c). However, at DPC concentrations of 0.25 g/L and 0.5 g/L, the SOD activities in cotton plants infested with aphids at low population density were significantly lower than in plants infested with aphids at high population density (Fig. 7c).

At 20th day, without aphids, the SOD activity of the 0.05 g/L DPC treatment was the highest, significantly higher (4.71%) than that of controls (*P* < 0.05; Fig. 6d), while the SOD activity at 0.5 g/L DPC was the lowest, significantly lower (13.13%) than that in the controls (*P* < 0.05; Fig. 6d). The SOD activity of cotton under low density changed markedly with increasing concentration of DPC, and reaching a maximum at 0.05 g/L DPC, significantly higher than that of cotton without DPC treatment (*P* < 0.05; Fig. 6d). However, the SOD activity difference at this DPC concentration was the most significant, and the SOD activity under low density was significantly lower (6.27%) than that of cotton without aphid (*P* > 0.05; Fig. 7d). The SOD activity of cotton under high density changed gently with increasing DPC concentration, the SOD activity was significantly higher than that of the controls only when the concentration of DPC was 0.05 g/L or 0.1 g/L (*P* < 0.05; Fig. 6d). When the DPC concentrations ≥ 0.25 g/L, the SOD activities in the low and high aphid population density groups were significantly higher than in the control (*P* < 0.05; Fig. 7d).

**Fig. 8:**
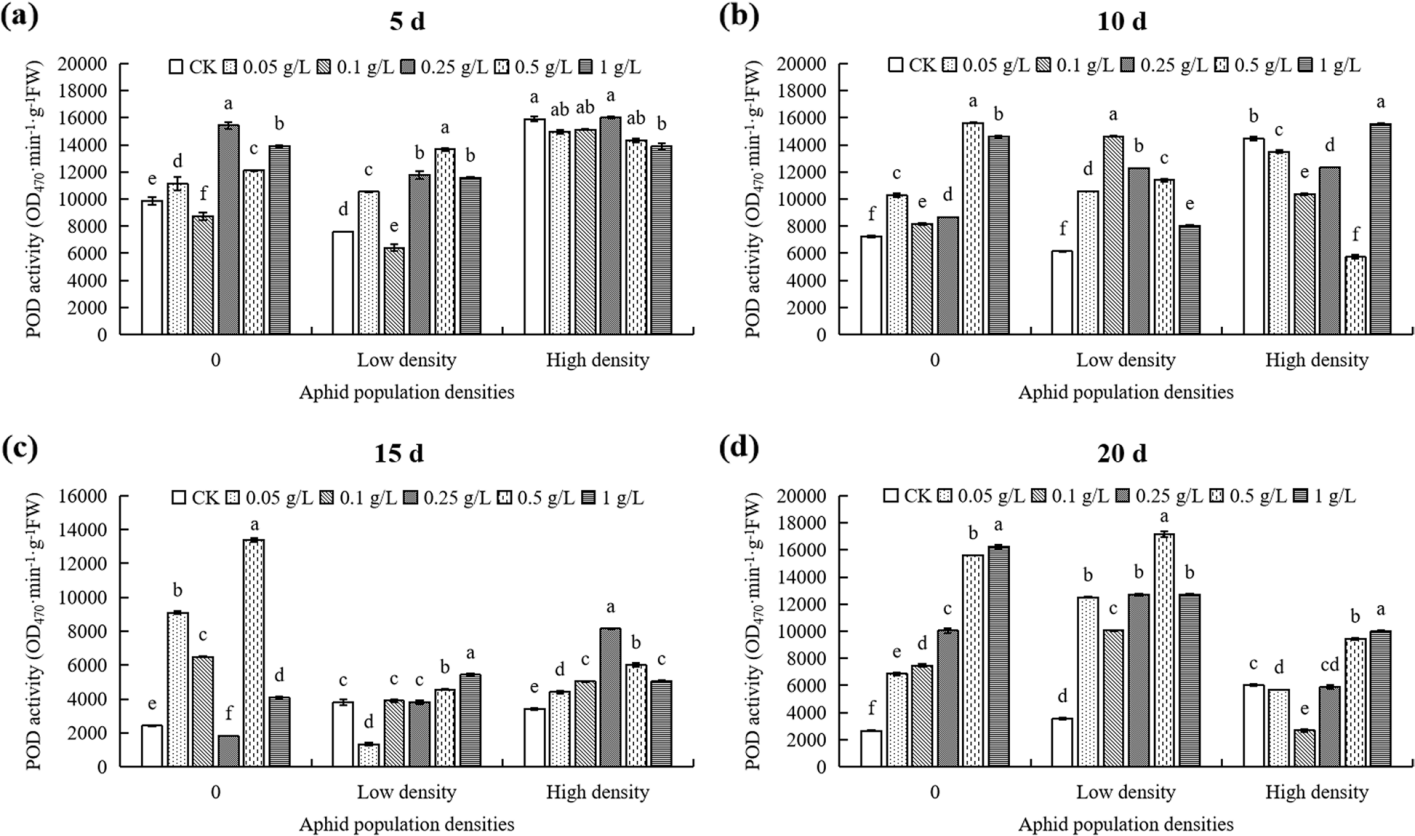
Effects of DPC treatment on POD (peroxidase) activity in cotton plants infested with aphids at three densities 5 (**a**), 10 (**b**), 15 (**c**), and 20 d (**d**) after spraying with DPC. Lower-case letters indicate significant differences between the control (CK) cotton plants and the plants in the five DPC treatments at the three aphid population densities

**Fig. 9:**
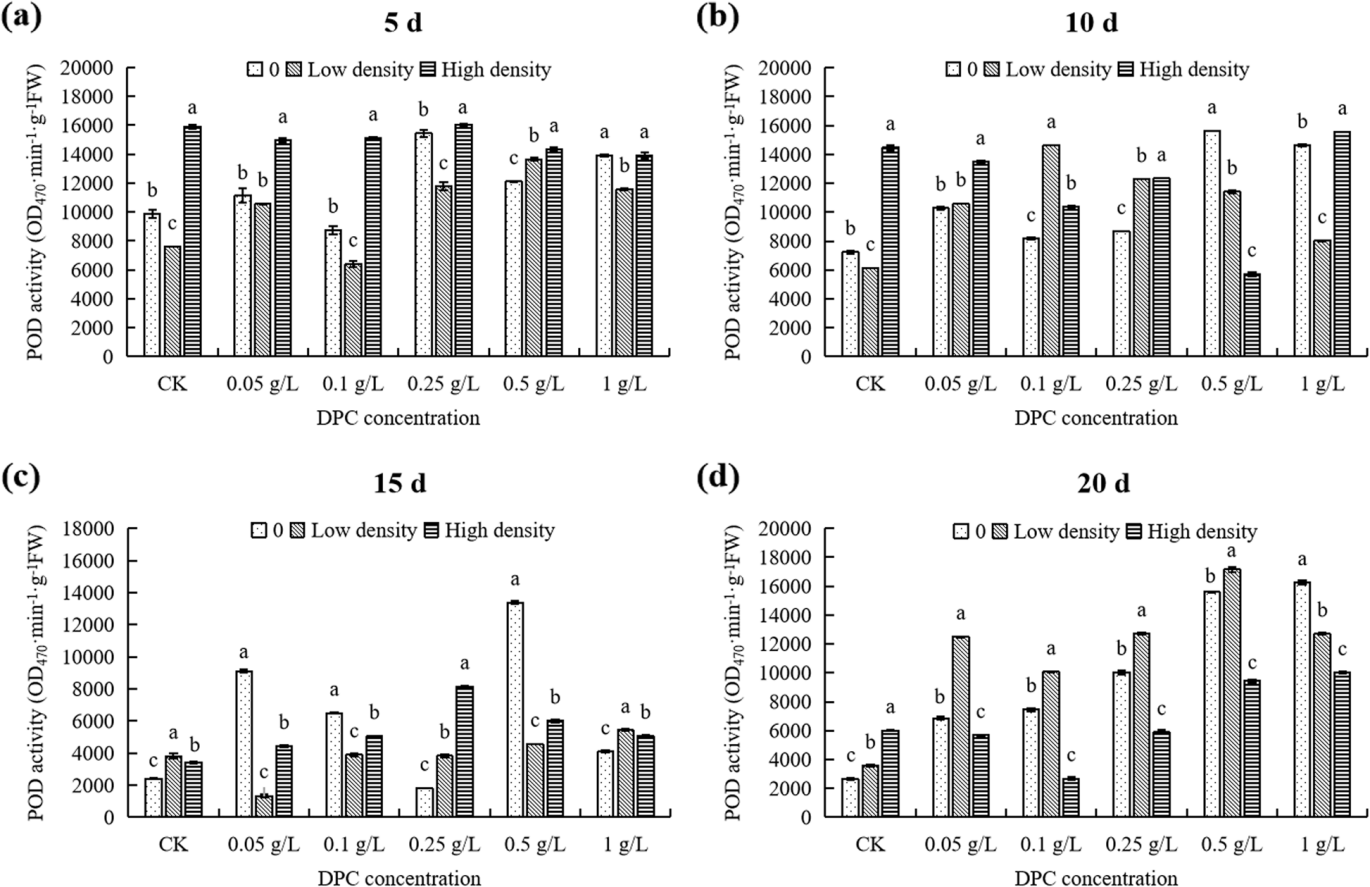
Effects of DPC treatment on POD (peroxidase) activity in cotton plants infested with aphids at three densities 5 (**a**), 10 (**b**), 15 (**c**), and 20 d (**d**) after spraying with DPC. Lower-case letters indicate significant differences between the cotton plants infested with aphids at three population densities for the five DPC concentrations

At 5th day, at low population density and 0.1 g/L DPC treatment, POD activity was the lowest (6400 OD_470_·min^−1^·g^−1^ FW) (Fig. 8a). Also, under high density, the POD activity reached the highest value at 0.25 g/L DPC, higher (62.54%) than in the for untreated controls (Fig. 8a). At 1 g/L DPC treatment, the POD activity at low density is significantly different from that at 0 and high aphid densities (*P* < 0.05; Fig. 9a). At DPC concentrations < 1 g/L, the POD activities at the under high density were highest for each concentration of DPC (Fig. 9a).

At 10th day, under low density, the POD activity reached the maximum at 0.1g/L,which significantly higher than the control (Fig. 8b). Under high density, the POD activity in cotton was the lowest at 0.5 g/L (5718.33 OD_470_·min^-1^·g^-1^ FW), and the POD activity in cotton reached the highest at 1g/L, which was 1.15-fold higher than in the control (Fig. 8b). Except the DPC concentration was 0.5 g/L, the POD activity in high density was higher than that in 0 density (Fig. 9b).

At 15th day, under low density, the POD activity of cotton increased with increasing DPC concentration, and the POD activity was lowest under 0.05 g/L DPC treatment, significantly lower (65.14%) than that of controls (Fig. 8c). Under high density, the POD activity of cotton first increased then decreased with increasing DPC concentration (Fig. 8c), and the POD activity was highest under treatment with 0.25 g/L DPC, 1.38-fold higher than without DPC treatment (Fig. 8c). At each DPC concentration, the effects of the different aphid densities on POD activity in cotton were significant (*P* < 0.05; Fig. 9c).

At 20th day, at 0 density, the POD activity in cotton was significantly different at the different DPC concentrations (Fig. 8d). Under low density, the POD activity reached the maximum value at 0.5 g/L DPC treatment, 3.83-fold higher than that without DPC treatment (Fig. 8d). When the DPC concentration was 0.1 g/L, the POD activity was lowest (2673.33 OD_470_·min^−1^·g^−1^ FW), significantly lower (55.53%) than in the control (Fig. 9d). When the DPC concentrations was 0.05–0.5 g/L, the POD activity in cotton with low density stress was significantly higher than that with high density (*P* < 0.05; Fig. 9d).

**Fig. 10:**
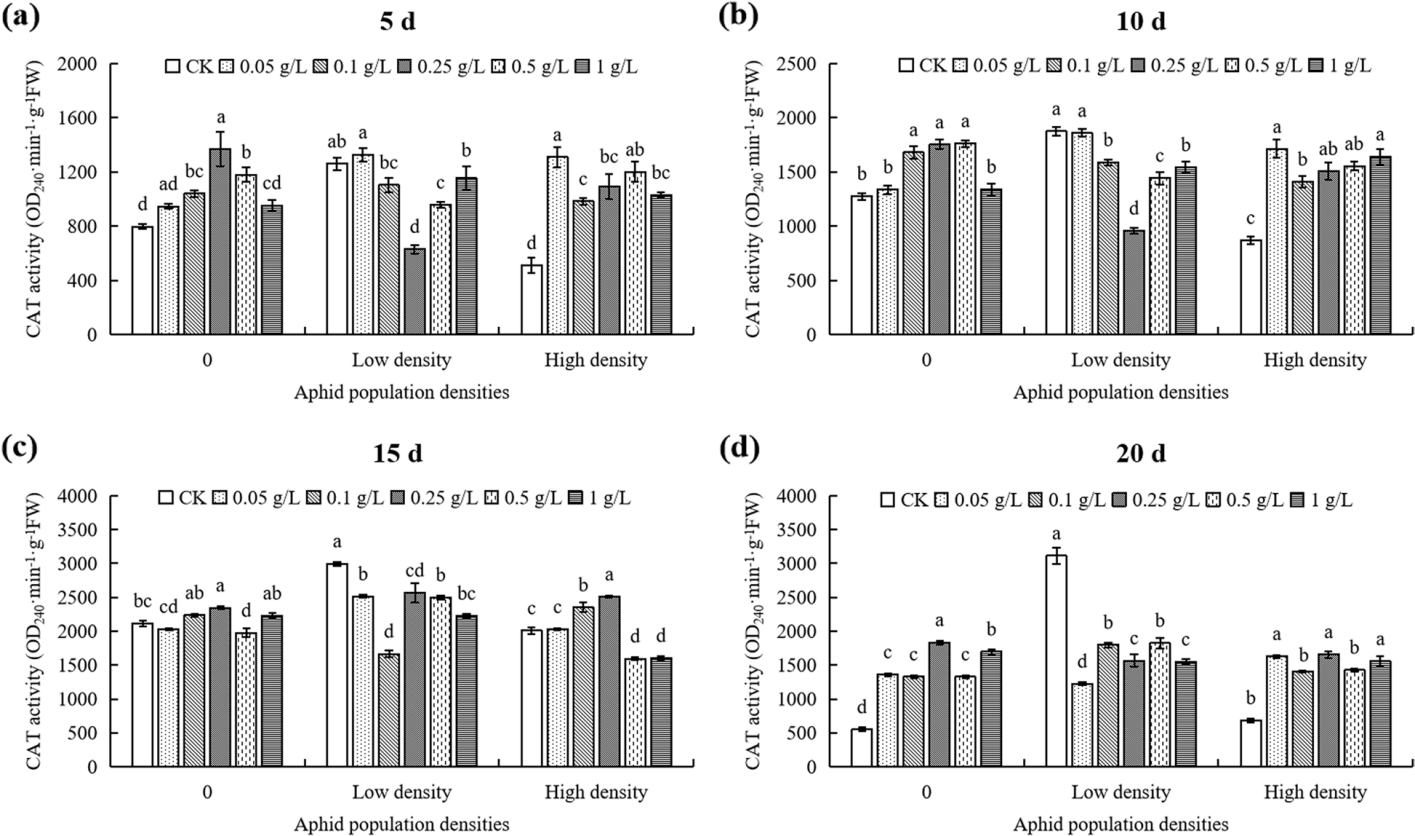
Effects of DPC treatment on CAT (catalase) activity in cotton plants infested with aphids at three densities 5 (**a**), 10 (**b**), 15 (**c**), and 20 d (**d**) after spraying with DPC. Lower-case letters indicate significant differences between the control (CK) cotton plants and the plants in the five DPC treatments at the three aphid population densities

**Fig. 11:**
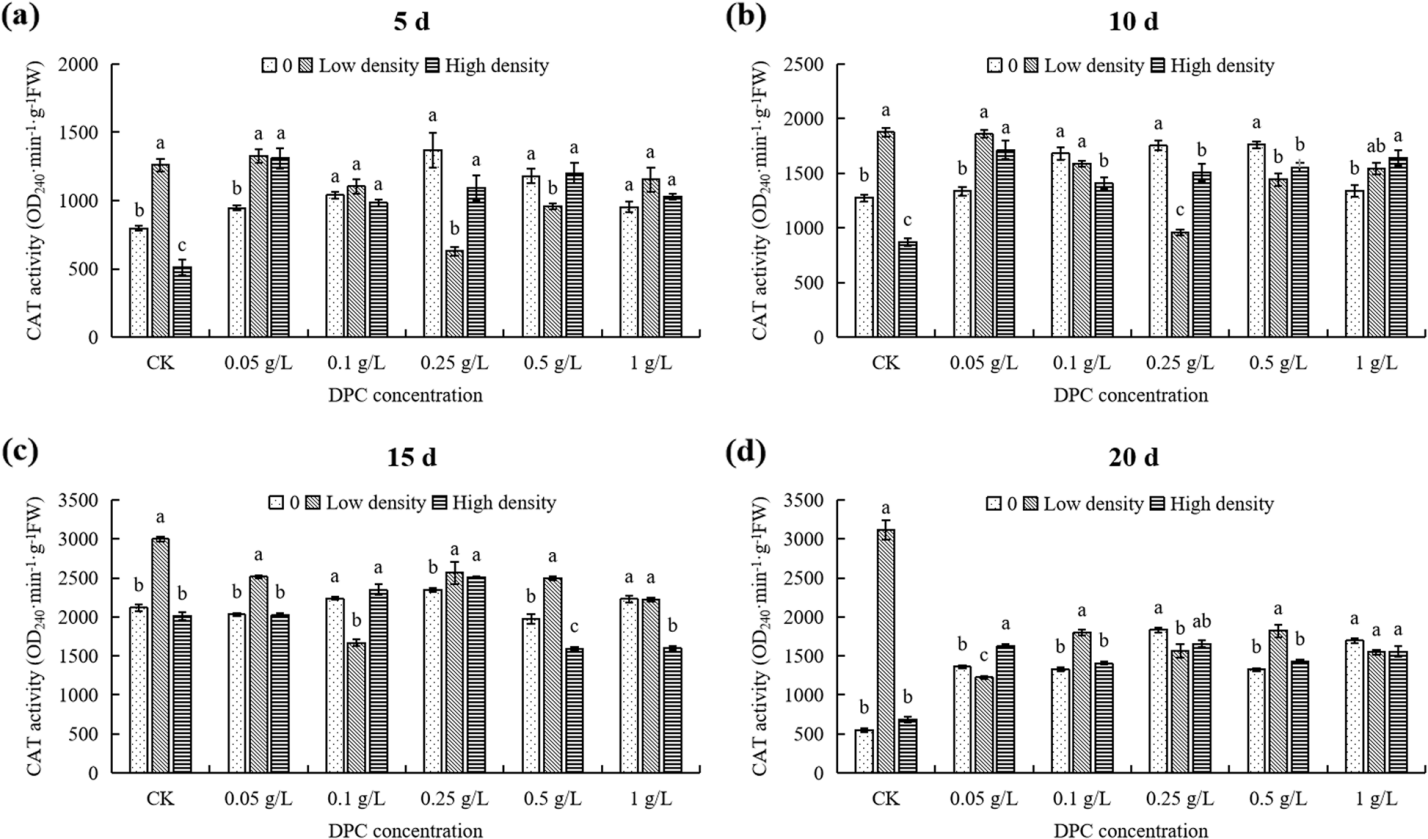
Effects of DPC (treatment on CAT (catalase) activity in cotton plants infested with aphids at three densities 5 (**a**), 10 (**b**), 15 (**c**), and 20 d (**d**) after spraying with DPC. Lower-case letters indicate significant differences between the cotton plants infested with aphids at three population densities for the five DPC concentrations

At 5th day, at 0 density, the CAT activity reached the maximum value at 0.25 g/L DPC, significantly higher (71.96%) than that of untreated controls (Fig. 10a). Under low density, the CAT activity reached the minimum value in the 0.25 g/L treatment, significantly lower (50%) than that in the control (Fig. 10a). At high density, the concentrations of DPC < 0.25 g/L, the CAT activities in cotton were significantly different from the activities in plants in the > 0.25 g/L DPC treatments (Fig. 10a). When the DPC concentration was 0 g/L (CK), the CAT activity in cotton with high density was significantly lower (35.93%) than low density (*P* < 0.05; Fig. 11a). When the DPC concentration was 0.25 g/L, the CAT activity in cotton with high density was significantly higher (35.93%) than low density (*P* < 0.05; Fig. 11a).

At 10th day, at 0 aphid density, the CAT activity reached the maximum value at 0.5 g/L, significantly higher (38.12%) that in the untreated plants (Fig. 10b). At low aphid population density, the CAT activity reached a minimum value at 0.25 g/L DPC, significantly lower (49.01%) than that of the control (*P* < 0.05; Fig. 10b). The CAT activities in cotton infested with aphids at high density were significantly different (*P* < 0.05; Fig. 10b). At DPC concentrations of 0.1, 0.25, and 0.5 g/L, the CAT activity in plants infested with aphids at both low and high density was lower than in the no-aphid control (Fig. 11b).

At 15th day, at 0 and low density, the CAT activities in cotton with different DPC concentrations were significantly effected than control (Fig. 10c). In plants infested with aphids at high density, the CAT activity reached the maximum value at 0.25 g/L DPC, significantly higher than in the controls (Fig. 10c), and it reached the minimum value at 0.5 g/L DPC, significantly lower (20.73%) than in cotton without DPC treatment (Fig. 10c). When the DPC concentration was 0.25 g/L, the CAT activity in cotton with low and high density were significantly higher than without aphids (*P* < 0.05; Fig. 11c).

At 20th day, the CAT activity in cotton under different densities was significantly different with different concentrations of DPC (Fig. 10d). Without aphids, the CAT activity without DPC treatment was the lowest (550 OD_470_·min^−1^·g^−1^ FW) (Fig. 10d). When the DPC concentration was 0 g/L, the CAT activity in cotton infested with aphids at low density was 24.18% higher than it was in plants with no aphids (*P* < 0.05; Fig. 11d), and the CAT activity of cotton infested with aphids at high density was 4.66-fold higher than that of 0 aphid (*P* > 0.05; Fig. 11d). When treated with 1 g/L DPC, the difference in CAT activity with population density was not significant (*P* > 0.05; Fig. 11d).

## Discussion

When plants are subjected to stress, the balance between the production and elimination of reactive oxygen species (ROS) is disrupted, and the elimination of ROS mainly relies on protective enzyme systems such as SOD, POD, and CAT, as well as other non-enzymatic systems [40]. Plant growth regulators can optimize cellular physiological and biochemical metabolism in plants, and enhance resistance by increasing the levels of protective enzymes [41, 42]. In the present study, spraying DPC at different concentrations enhanced the soluble protein content and POD and CAT activities in plants (Fig. 1a, c and d). When the DPC concentration was ≤ 0.1 g/L, the SOD activity also showed an increasing trend (Fig. 1b). However, after 20 days of DPC treatment, the SOD and CAT activities in cotton showed opposite trends compared with those in the early stage of the experiment (Fig. 1b and d), which might be due to a gradual decrease in the efficacy of DPC over time.

DPC enhanced cotton resistance through a variety of ways. For example, DPC enhances cotton resistance by promoting root growth [43, 44]. Furthermore, DPC enhances photosynthesis in cotton leaves by increasing the chlorophyll content, photosynthetic rate, and the relative chlorophyll content (SPAD value) [25, 26]. DPC also improves cell membrane stability by regulating ions salts such as Na^+^, K^+^, and Cl^−^ [22, 23], enhancing cell osmotic pressure resistance by increasing the proline and protein content in cotton [24, 25], and enhancing secondary metabolites of cotton by increasing phenolic acids such as lignin, total phenols, and tannins [27]. The present work showed that DPC enhanced stress resistance by increasing the metabolic levels of protective enzymes such as SOD, POD, and CAT in cotton. We found that in response to the stress of cotton aphids, cotton itself increases the activity of soluble protein and protective enzymes to defend against cotton aphids, but this defense reaction will be weakened with the high denisty and continued harm of cotton aphids (Figs. 4, 5, 6, 7, 8, 9, 10 and 11). After DPC treatment, the soluble protein content and protective enzymes activity were significantly increased, and the duration of defense was also prolonged (Figs. 4, 5, 6, 7, 8, 9, 10 and 11). We think that this is a good phenomenon, which can help cotton to defend against high density aphids and prolong the duration of defense.

DPC not only regulates the growth of cotton; it also affects insect pests feeding on cotton. For example, when DPC was applied in the field, the relative survival number of 100 larvae of *Helicoverpa armigera* decreased by 28.7% compared with blank controls, the damage rates of top and bud bolls decreased by 6.6% and 11.1% respectively [45], and the reproduction rate of spider mites decreased by 45.7% and 57.33% [30]. In order to prevent DPC from affecting themselves, insects activate metabolic detoxification enzymes such as GST and CarE to degrade exogenous toxins and maintain normal physiological metabolism [46]. In the present study, field and laboratory experiments showed that DPC could significantly increase GST activity in cotton aphids (Figs. 2a and 3a), but the activities of CarE and AchE were downregulated (Figs. 2b, c and 3b, c), indicating that DPC was also toxic to cotton aphids during application to cotton fields, presumably by altering detoxification enzyme activities in cotton aphids, and the main target of DPC in cotton aphids was GST.

Plant growth regulators affect all aspects of plant growth, and plant–insect interactions are also likely to be affected [47]. During the growth period of cotton, the use of DPC also affected the interaction between cotton and cotton bollworm. DPC induces cotton to produce more tannins and terpenoids, both of which are related to insect resistance to cotton [48]. DPC also causes the cotton bollworm to resist feeding, and reduces its growth and survival rates, thereby reducing the harmfulness of cotton bollworm to cotton [47]. Our findings are similar in that DPC also affected this relationship in a cotton-cotton aphid system. Regarding damage by different densities of cotton aphids, DPC positively responded to cotton aphid stress by inducing the production of protective enzymes and proteins in cotton (Figs. 4, 6, 8 and 10), and enhanced the resistance of cotton to aphids.

Increased use of DPC can enhance stress resistance in many plants. In the growth process of peanut and sunflower, application of DPC can reduce the MDA content and plant height, increase the activity of protective enzymes, and improve stress resistance [49, 50]. Similarly, spraying DPC on winter barley, rape, and maize can reduce plant height, improve stem physical strength, and enhance plant lodging resistance [51, 52]. DPC enhances the drought resistance of Eucalyptus tree species by promoting root growth [53]. In cotton, DPC can inhibit the occurrence of some pests and diseases, such as cotton bollworm, spider mites, and cotton verticillium wilt [29, 30, 54, 55]. Our results show that DPC can increase the tolerance of cotton to aphids (Figs. 4, 5, 6, 7, 8, 9, 10 and 11). However, in the face of different external pressures, the mechanism by which DPC improves plant resistance and adaptation requires further investigation.

## Conclusion

In summary, the results of our study showed that DPC stimulated the protective enzymes defense mechanism in cotton in the cotton-cotton aphid system. We found that DPC treatment increased soluble protein content and the activities of the protective enzymes in cotton. At the same time, DPC also had a direct toxic effect on cotton aphids by targeting GST, which had a positive effect on alleviating the responses of cotton to cotton aphid stress. Finally, we compared the protective enzyme activity in cotton before and after feeding by cotton aphids, and further confirmed that DPC can induce protective enzymes activity in cotton to defend against the stress caused by cotton aphid feeding. Therefore, we conclude that DPC interferes with cotton aphids through indirect (DPC induced cotton defense responses) and direct (DPC toxicity to cotton aphids) ways, which plays a positive role in interfering with cotton aphids. However, the mechanism by which DPC mediates cotton defenses against cotton aphids requires further investigation to identify the genes/proteins, pathways, and regulatory aspects involved.

## Data Availability

The raw data of the presented results of this study are available on request to the corresponding author.

## Abbreviations

DPC: Mepiquat chloride
GST: Glutathione S-transferase
CarE: Carboxylesterase
AchE: Acetylcholinesterase
POD: Peroxidase
CAT: Catalase
SOD: Superoxide dismutase
MDA: Malondialdehyde
